# Quality of Life Among Down Syndrome Patients With and Without Congenital Heart Disease at King Abdulaziz University Hospital, Jeddah, Saudi Arabia

**DOI:** 10.7759/cureus.33553

**Published:** 2023-01-09

**Authors:** Fatimah A Alhaddad, Naif A Alkhushi, Amal M Alharbi, Sarah A Al Talib, Sarah M Sultan, Yara O Bahawi

**Affiliations:** 1 Medicine, King Abdulaziz University Faculty of Medicine, Jeddah, SAU; 2 Department of Pediatrics, Ministry of National Guard Health Affairs, King Abdulaziz Medical City, Jeddah, SAU; 3 Pediatric Cardiology, King Abdulaziz University Faculty of Medicine, Jeddah, SAU

**Keywords:** congenital heart disease, congenital heart defect, trisomy 21, down syndrome, quality of life

## Abstract

Background

Congenital heart diseases (CHD) are common in Down syndrome patients who will often have additional anomalies, in which the presence of them and their management are expected to impact their quality of life (QoL). There are limited studies trying to evaluate the impact of CHD on the QoL in children with Down syndrome.

Methods

The present study comprised 97 Down syndrome children. The children’s parents responded to phone interviews filling out TNO-AZL (Netherlands Organisation for Applied Scientific Research Academic Medical Centre) Preschool Quality of Life (TAPQOL) and TNO-AZL Child Quality of Life Parent Form (TACQOL-PF) questionnaires. Children were divided into two groups according to their age: group A (one to five years) and group B (six to 15 years). The results were analyzed using Statistical Package for Social Sciences (SPSS) software, version 21 (IBM Corp., Armonk, NY).

Results

CHD negatively affected motor skills in younger but not older children. All other QoL-related parameters were unaffected by CHD.

Conclusion

Down syndrome children with CHD demonstrated similar QoL to Down syndrome children without CHD, with the exception of having a lower motor outcome as infants/toddlers. This difference improved with time and did not exist in older children.

## Introduction

Down syndrome (trisomy 21) is the most common autosomal abnormality in infants, with a prevalence of one in 500 live births in the absence of prenatal screening [1]. It is the main genetic cause of intellectual disability in humans, impairing attention, learning, memory, and language. It also delays cognition and the development of motor skills. As such, it reduces the child’s ability to interact with the environment, explore space, and manipulate objects [2,3].

The distinctive facial features of the Down syndrome phenotype make it an apparent disability, which may hinder interpersonal relationships and encourage isolation [4]. Other congenital abnormalities resulting from the presence of the extra chromosome include thyroid abnormalities, gastrointestinal disorders, delayed growth, and congenital heart disease (CHD) [3,5-7]. CHD is a major cause of morbidity and mortality in patients with Down syndrome [8], with an incidence of approximately 86.8% in Saudi Arabia compared with 42-44% worldwide, with the most predominant type being patent ductus arteriosus (PDA) (47.8%) [3,9-11].

Some patients with Down syndrome develop secondary acquired conditions, such as autoimmune alopecia, sleep problems, leukemia, respiratory infections, and pulmonary hypertension [12-16], all of which limit daily activities and profoundly diminish the quality of life (QoL) [17,18]. Assessment of QoL provides an insight into a person’s perception of his/her feelings of contentment in daily functioning and in different aspects of life (e.g., physical, material, social, and psychological) [19-21]. In theory, CHD negatively affects the QoL of any patient and his/her family, as it is associated with a high comorbidity rate [22].

In a previous study, children with Down syndrome had worse health-related QoL (HRQoL) scores on the gross motor skills, autonomy, and social and cognitive functioning scales than normal children. Surprisingly, their scores on the physical complaints scale indicated no major issues [23]. In contrast, in another study, the mean scores for autonomy were within the normal range, as were those for psychological well-being, parent relations, and school environment. However, the mean scores on the physical well-being, social support, and peers scales were lower in the Down syndrome group than in the control group. Moreover, adolescents with Down syndrome scored lower on all scales than younger children with Down syndrome [18].

Studies assessing QoL in pediatric and adolescent patients with Down syndrome and CHD are limited both worldwide and in Saudi Arabia. This study provides important insights into the potential effect of CHD on the QoL of children with Down syndrome.

## Materials and methods

The study was conducted from June to August 2020 at the Pediatric Department of King Abdulaziz University Hospital (KAUH), Jeddah, Saudi Arabia. It was approved by the Institutional Review Board of KAUH (reference number: 324-20). Informed consent was obtained from the parents of all participants before distributing the questionnaires.

Our study compared QoL in Down syndrome children with and without CHD. The medical records of Down syndrome patients aged between one and 15 years were obtained from KAUH with the following International Classification of Diseases, Tenth Revision (ICD-10) codes: Q90.0, Q90.1, and Q90.9. Age, gender, nationality, CHD type, and chronic illnesses were extracted from the database. All of our patients were diagnosed prenatally, upon birth, or within the first year of life clinically, and their diagnosis was confirmed by genetic testing. Patients who were hospitalized during the data collection period, whose parents refused to participate or registered with a wrong phone number, or who were deceased were excluded. The patients were divided into two groups according to their age: group A (one to five years) and group B (six to 15 years), as is done in the available standardized QoL tests.

The data were collected by having the parents respond to questions via phone interviews. Two questionnaires were used: the TNO-AZL (Netherlands Organisation for Applied Scientific Research Academic Medical Centre) Preschool Quality of Life (TAPQOL) questionnaire for children aged one to five years [24,25] and the TNO-AZL Child Quality of Life Parent Form (TACQOL-PF) for children aged six to 15 years [26,27]. The questions were in Arabic or English and explained to the parents, if needed. The researchers filled out a questionnaire based on parent responses.

TAPQOL

The TAPQOL questionnaire is a generic, multidimensional instrument that uses 43 items to assess HRQoL in four domains divided into 12 subdomains. The four domains are as follows: physical functioning (sleeping; appetite; lung, stomach, and skin problems; and motor functioning), social functioning (problem behavior), cognitive functioning (communication), and emotional functioning (anxiety, positive mood, and liveliness). The number of items per scale ranged from three to seven. There is no overall summary score, but rather a series of domain scores, each scored between 0 and 100. Higher scores indicate better QoL.

TACQOL-PF

The TACQOL-PF consists of 56 items divided into seven domains: physical complaints (pain and symptoms), motor functioning, cognitive functioning, autonomy, social functioning, and positive and negative emotional functioning. Physical complaints included those related to motor functioning, autonomy, cognitive functioning, and social functioning. Each item in this domain has two linked questions: the first addresses the frequency of the complaint in the past few weeks, and the second focuses on how the child felt about the complaint. The overall scores were calculated by summing the item scores in each scale, with higher scores indicating better QoL.

Data entry and analysis

The data were placed in Microsoft Excel 2020 (Microsoft Corporation, Redmond, WA) and analyzed using Statistical Package for Social Sciences (SPSS) software, version 21 (IBM Corp., Armonk, NY). Participant characteristics were assessed in terms of frequency and central tendency. The TAPQOL and TACQOL-PF scores were compiled in a syntax file created by the authors. Missing scores were handled according to the guidelines of the questionnaire. Scores were calculated from all answered items. A score was declared missing if more than two items in a given scale were left unanswered.

An independent sample t-test was used to compare the QoL scores in the stratified analysis for nationality, the presence of CHD, and the presence of a chronic condition. Pearson’s correlation was used to compare the domain scores in both questionnaires. P-values < 0.05 were considered significant. All tests compared age-matched patients with and without CHD.

## Results

Among the 162 children with Down syndrome at our hospital, 97 met the inclusion criteria (of whom 54 were Saudi and 43 non-Saudi); 65 were excluded because their parents refused to participate (n = 9) or could not be reached (n = 56). The patients were divided into two groups according to their age: group A (one to five years of age) and group B (six to 15 years of age).

Group A (one to five years of age)

Group A comprised 57 children, 40% of whom were boys. The detailed demographic data for this group are shown in Table 1. Among the 57 cases, 42 had CHD, which was classified as follows: atrioventricular septal defect (AVSD, 40.5%), large ventricular septal defect (VSD, 23.8%), PDA (14.3%), small VSD (7.1%), small PDA (4.8%), tetralogy of Fallot (TOF, 2.4%), coarctation of the aorta (COA, 2.4%), pulmonary atresia VSD (2.4%), and AVSD-TOF (2.4%). Additional chronic problems were present in 61.9% of the patients with CHD and 80% of those without CHD. There was no significant difference between the CHD subgroups in terms of the distribution of each major category of chronic illnesses.

**Table 1:** Group A (one to five years of age) characteristic CHD: congenital heart disease.

	CHD	Non-CHD	P-value
Number of subjects	42 (73.7%)	15 (26.3%)	0.43
Age in years (mean ± SD)	2.7 ± 1.5	3 ± 1.2	0.37
Male gender	23 (54.8%)	10 (66.7%)	0.42
Chronic diseases	26 (62%)	12 (80%)	0.21
A. Hypothyroidism	19 (45.2%)	7 (46%)	0.93
B. Gastrointestinal tract abnormality	3 (7.1%)	0 (0%)	0.95
C. Cataract	2 (4.8%)	2 (13.3%)	0.27
D. Gastroesophageal reflux	2 (4.8%)	1 (6.7%)	0.80
Other chronic diseases	14 (33.3%)	7 (46%)	0.39

The CHD and non-CHD subgroups had low scores in the communication (68.9 vs. 68.3) and problem behavior (69.2 vs. 69) domains of the TAPQOL questionnaire. The differences between the subgroups were not significant.

Children with CHD had significantly worse motor function than those without CHD (mean score: 80.4 vs. 92.7, P = 0.02). In all other domains, the overall score was >82 for the CHD subgroup and >87 for the non-CHD subgroup. All individual domain scores were lower (although not statistically significant) in the CHD subgroup. Table 2 details the comparisons between the subgroups.

**Table 2:** TAPQOL results TAPQOL domain scores among children aged one to five years of age. TAPQOL: TNO-AZL Preschool Quality of Life; CHD: congenital heart disease.

Scale	CHD mean (SD)	Non-CHD mean (SD)	P-value
Stomach	82.9 (24.8)	92.8 (14)	0.069
Skin	89.7 (20.6)	87.2 (18.3)	0.684
Lung	88.5 (22.6)	90.6 (20.6)	0.758
Sleep	85.6 (16.5)	92.1 (11.9)	0.168
Appetite	89.6 (13.7)	90.6 (15.1)	0.829
Liveliness	92.1 (21.2)	97.8 (8.6)	0.155
Positive mood	92.9 (16.1)	97.8 (8.6)	0.147
Problem behavior	69.2 (27.9)	69 (18)	0.983
Anxiety	82.1 (29.1)	92.2 (15.3)	0.098
Social functioning	86.9 (29.7)	95.2 (13.8)	0.320
Motor functioning	80.4 (16.9)	92.8 (13.2)	0.024
Communication	68.9 (17.9)	68.3 (18.7)	0.913

When stratified for nationality (Saudi and non-Saudi), non-Saudi children with CHD had lower scores in the stomach, lung, and sleep problems and positive mood domains than did those without CHD (P = 0.00, 0.01, 0.01, and 0.02, respectively). In contrast, CHD had no significant effect on the QoL domain scores for Saudi children.

Group B (six to 15 years of age)

Group B comprised 40 children, 62% of whom were boys. The detailed demographic data for this group are shown in Table 3. Among the 40 cases, 25 had CHD, which was classified as large VSD (36%), AVSD (20%), atrial septal defect (ASD) (16%), small VSD (12%), small PDA (8%), and large PDA (4%). None of the patients in group B had TOF, COA, PDA-VSD, or AVSD-TOF. Additional chronic problems were present in 76% of the patients with CHD and 40% of those without CHD. Significantly, more children in the CHD vs. non-CDH subgroup had hypothyroidism (P = 0.01). There was no significant difference in the distribution of the remaining chronic conditions between the subgroups.

**Table 3:** Groups B (six to 15 years of age) characteristics CHD: congenital heart disease.

	CHD	Non-CHD	P-value
Number of subjects	25 (62.5%)	15 (37.5%)	0.20
Age in years (mean ± SD)	9.68 ± 2.6	9.67 ± 2.2	0.44
Male gender	17 (68%)	8 (53%)	0.35
Chronic diseases	19 (76%)	6 (40%)	0.03
A. Hypothyroidism	13 (52%)	2 (13.3%)	0.01
B. Gastrointestinal tract abnormality	2 (8 %)	2 (13.3%)	0.62
C. Gastroesophageal reflux	1 (4%)	2 (13.3%)	0.35
D. Coeliac disease	3 (12%)	2 (13.3%)	0.91
Other chronic diseases	13 (52%)	4 (26.7%)	0.12

None of the scores in the TACQOL-PF domains differed significantly between the CHD and non-CHD subgroups, as shown in Table 4. Both subgroups had low scores in cognitive functioning and emotional domains. Not as the younger age group, nationality had no effect on the HRQoL for both the CHD and non-CHD subgroups.

**Table 4:** TACQOL results TACQOL domains scores among six to 15 years of age children. TACQOL: TNO-AZL Child Quality of Life; CHD: congenital heart disease.

Scale	CHD mean (SD)	Non-CHD mean (SD)	P-value
Physical domain	28 (4.8)	27.3 (5.4)	0.644
Motor domain	26.5 (4.9)	26.1 (4.5)	0.771
Autonomy	27 (4.5)	25.5 (5.9)	0.352
Cognitive functioning	22.8 (4.5)	19.9 (8.1)	0.212
Social functioning	26.2 (3.2)	26.6 (4.5)	0.721
Positive emotions	13.5 (3.6)	13.9 (3)	0.694
Negative emotions	11.8 (2.6)	10.9 (3.2)	0.319

## Discussion

The results of this study contradict the assumption that CHD significantly impacts QoL in children with Down syndrome. The only exception was in motor function, which was worse in children with vs. without CHD aged one to five years. However, as these children grow up, this difference was no longer apparent. The low motor function scores in younger children with CHD could be attributed in part to frequent hospital admissions, prolonged hospitalization after cardiac surgery, and lack of stimulation for perceived cardiovascular limitations by caregivers [28]. They may also be the outcome of cardiac surgery, as is the case for children without Down syndrome [29,30].

Improved motor function with age was also observed in other studies of children with Down syndrome. These include the study by Visootsak et al. (2011) [31], which compared children with AVSD vs. normal hearts, and also the study by Weijerman (2011) [32]. Interestingly, the latter study showed that communication skills deteriorated with age and that QoL was influenced by respiratory and gastrointestinal tract problems but not by CHD. Additionally, Alsaied et al. (2016) reported that infants and toddlers with Down syndrome and CHD had worse neurodevelopmental outcomes than did their non-CHD counterparts; however, this difference disappeared upon reaching school age [33].

Although numerous studies have shown that children with Down syndrome have a less favorable QoL than normal children; however, there is limited research on QoL in children with Down syndrome and CHD. Other than motor function, QoL in children with Down syndrome does not appear to be significantly affected by CHD, i.e., the QoL profiles of Down syndrome children with/without CHD are similar. Children with Down syndrome have less emotional expression, are less reactive vocally, and less responsive to their environment than the normally developing children [34]. Therefore, small non-verbal differences may pass unnoticed with standard assessment.

In our study, the scores in the negative emotions domain were quite low in group B regardless of whether CHD was present. This may reflect the performance of the study during the coronavirus disease 2019 (COVID-19) pandemic. Parents have reported that their child’s QoL noticeably improved when the child spend more time with their families and less time outdoors, and consequently, were less prone to respiratory diseases or infections. They also noticed that their children were more aggressive, short-tempered, and angry during vs. before the COVID-19 quarantine. In other words, the pandemic exacerbated their children’s negative emotions [35].

There was a moderate positive correlation between the scores on the lung problems and sleep scales in group A in our study. According to multiple studies, sleep-disordered breathing is the most common respiratory disorder in children with Down syndrome, with a prevalence ranging from 30% to 75% compared with 2% in normally developing children. Several studies have shown that sleep apnea in children with Down syndrome is often due to midfacial and mandibular hypoplasia, macroglossia, tonsillar and adenoidal hypertrophy, and generalized hypotonia [36-39].

In our analysis of group A, CHD significantly worsened QoL in non-Saudi children but did not affect QoL in Saudi children. However, this finding is based on a small number of subjects and hence requires further investigation. There are numerous social support and rehabilitation programs with subsidized costs for Saudi children, whereas those for non-Saudi children in low-income households are limited. This finding additionally may suggest that the observed difference in younger children may be confounded by nationality or income level. It will be interesting to study this in homogenous groups or with enough sample size to control for confounders, suggesting a better level of confidence.

Limitations

The study is limited by its relatively small sample size. However, the sample size was comparable to those in most previous studies of children with Down syndrome and CHD. Additionally, the family’s socioeconomic status was not taken into consideration. This may have affected the children’s QoL as it reflects the feasibility of their participation in special programs that might improve their QoL.

## Conclusions

The presence of CHD was associated with impaired motor function in younger but not older children with Down syndrome. Hence, motor function improves with age. This finding has important implications for health professionals and parents, as it highlights the need for more efficient physical therapy programs to achieve more favorable motor development outcomes.

None of the other QoL parameters were significantly affected by the presence of CHD. We suggest that the neurodevelopmental and QoL profiles are more affected by inherent genetic and mental disabilities and chronic respiratory and gastrointestinal problems than by CHD. Further studies with larger populations are needed to gain insights into the effect of different chronic diseases on the QoL of children with Down syndrome.
